# A community service review of the quality of inpatient discharge summaries from six inpatient wards at St Charles Hospital: an initial audit and quality improvement recommendations

**DOI:** 10.1192/bjo.2021.549

**Published:** 2021-06-18

**Authors:** Omar Mahmoud, Jasna Munjiza, Jacob King

**Affiliations:** 1 ST5 General Adult psychiatry Trainee, Pall Mall, North Kensington and Chelsea CMHT; 2 Consultant General Adult psychiatrist, Pall Mall, North Kensington and Chelsea CMHT; 3 Core trainee, Pall Mall, North Kensington and Chelsea CMHT

## Abstract

**Aims:**

To discuss whether Discharge summaries include important information to community mental health teams .

To identify patterns and produce recommendations for change by Quality improvement methods .

**Method:**

A convenience sample was selected of the first 5 patient discharges from each of the 6 adult inpatient wards at St Charles Hospital. This represented a total of 30 reviewed summaries. Outcome items were generated following discussion with community psychiatric colleagues based on those aspects of an admission thought to be of most use to a community mental health team. These were; reason for admission, diagnosis, circumstances of admission, progress on the ward, risk assessment, physical health, legal status on discharge, discharge medication, discharge management plan, contact details. Basic identification was also recorded as was the ward and date of discharge

**Result:**

- Only 3.3% (1/30) of discharge summaries were complete of all items.
- However 23.3% (7/30) were almost complete, failing to record only a single item, and a further 2 missing only 2 of 10 items. There was a bimodal distribution (Graph 1).
- Seven (7/30) discharge summaries provided no information. Of these, four (4/7) discharge summaries were written in the progress notes directly, rather than using the discharge summary proforma.
- The ‘reason for admission’ item was a clear low outlier with only 2/30 reporting this piece of information. For a number of cases, this was recorded unhelpfully as “in crisis”.

**Conclusion:**

There was limited evidence of systemic patterns,however some wards showed internal stark differences with some summaries complete or almost complete and others empty.

The key findings from this report are the high number of discharge summaries which have either no responses to them (7/30). This may indicate that the writer did not know how to use the current discharge template, and therefore support with using this is indicated. For those with a very low (7/30) number of item responses, we might conclude that these discharge summaries were written by someone with knowledge of using the system, but for another reason did not complete the majority of the items asked, and for this reasons are not immediately clear. Similarly, as highlighted above the main low outlying result relates to the apparent widespread practise of writing “in crisis” as the ‘reason for admission’, unfortunately to community teams this is an unhelpful and self-evident response.

